# Incidence, clinical risk and prognostic factors for liver metastasis in patients with cervical cancer: a population-based retrospective study

**DOI:** 10.1186/s12885-021-08127-6

**Published:** 2021-04-16

**Authors:** Hang Liu, Xiangsen Ye, Di Li, Qian Yao, Yan Li

**Affiliations:** 1 Department of Clinical Laboratory, Renmin Hospital of Wuhan University, Jiefang Road 238, Wuhan, 430060 China; 2 Department of Clinical Laboratory, The Central Hospital of Wuhan, Tongji Medical College, Huazhong University of Science and Technology, Wuhan, China

**Keywords:** Cervical cancer, Liver metastasis, Incidence, Risk factor, Prognosis

## Abstract

**Background:**

Cervical cancer is one of the most frequent malignancies in women, particularly metastasis resulting in a poor prognosis. However, the clinical characteristics of cervical cancer patients with advanced liver metastasis have not been well investigated. We aimed to evaluate the incidence, clinical risk and prognostic factors for hepatic metastasis in cervical cancer patients.

**Materials and methods:**

The clinical features of patients diagnosed with cervical cancer were collected from the Surveillance, Epidemiology and End Result (SEER) public cancer database between 2010 and 2015. Multivariate logistic and Cox regression models were performed to identify potential risk and prognostic factors for liver metastasis in patients with cervical cancer.

**Results:**

A total of 431 patients (2.32%) developed liver metastasis in our analysis. The following characteristics were significantly associated with the development of liver metastasis: black ethnicity, uninsured status, higher tumor stage, poorer differentiated grade, non-squamous histology, non-surgery of primary site, patients with any additional lung, bone, and brain metastasis. Multivariate Cox regression showed that patients with additional lung metastasis, without radiotherapy, and without chemotherapy were negatively correlated with overall survival. Concurrent chemotherapy and radiotherapy was a favorable prognostic factor to improve overall survival, and chemotherapy showed to increase cause-specific survival. Additional lung metastasis was an independent characteristic for both risk and prognostic factors for hepatic metastasis in patients with cervical cancer.

**Conclusion:**

Our results found several potential clinical features that may be used to assess the risk and prognosis of liver metastasis in patients with cervical cancer. These associated factors may provide clinical indications for the early identification and treatment of cervical cancer patients with hepatic metastasis.

## Introduction

Cervical cancer is the second most frequent female genital system malignant tumor, causing approximately 30, 000 cancer-related deaths worldwide in 2018 [1]. The Surveillance, Epidemiology, and End Results (SEER) database estimated 4290 deaths due to cervical cancer in 2020 across the United States. HPV vaccination and cervical cancer routine screening have reduced the incidence and mortality of cervical carcinoma in some developed countries [2, 3]. However, advanced cervical cancer patients with distant metastases remain difficult to diagnose and treat. Besides presenting poor outcomes, distant metastasis severely reduces the quality of life of late-stage patients [4]. Among these, the median survival time of patients with single distant organ metastasis is only approximately 8 months, and decrease to 5 months when multiple metastases are detected [5].

The liver is a hematogenous metastatic site, following lung and bone, in cervical cancer patients [6]. Hepatic metastasis was also positively associated with worse overall survival [7]. Recently, studies based on the SEER database have identified risk factors for lung and bone metastasis after the initial diagnosis of cervical cancer. These risk factors included age, African American ethnicity, unmarried status, higher stage and grade tumors, histology type of non-squamous or adenocarcinoma [8, 9]. However, population-based studies to assess the clinical indicators of liver metastasis in cervical cancer patients remain scarce, probably because such patients are relatively rare.

Management of liver metastasis in patients with cervical cancer is similar to that of other metastatic organs. The main treatments usually consist of systemic chemotherapy combined with local radiotherapy, and with/without metastasectomy according to the patients’ condition [10]. The application of chemoradiation has been confirmed to prolong the overall survival time of cervical cancer patients with lung and bone metastases [4]. However, limited large-population researches have focused on the impact of this treatment model and other potential factors on the prognosis of cervical cancer patients with liver metastasis. In the present study, we used the SEER cancer database to identify the incidence, clinical risk and prognostic factors associated with liver metastasis in cervical cancer patients.

## Materials and methods

### Study design

**Fig. 1:**
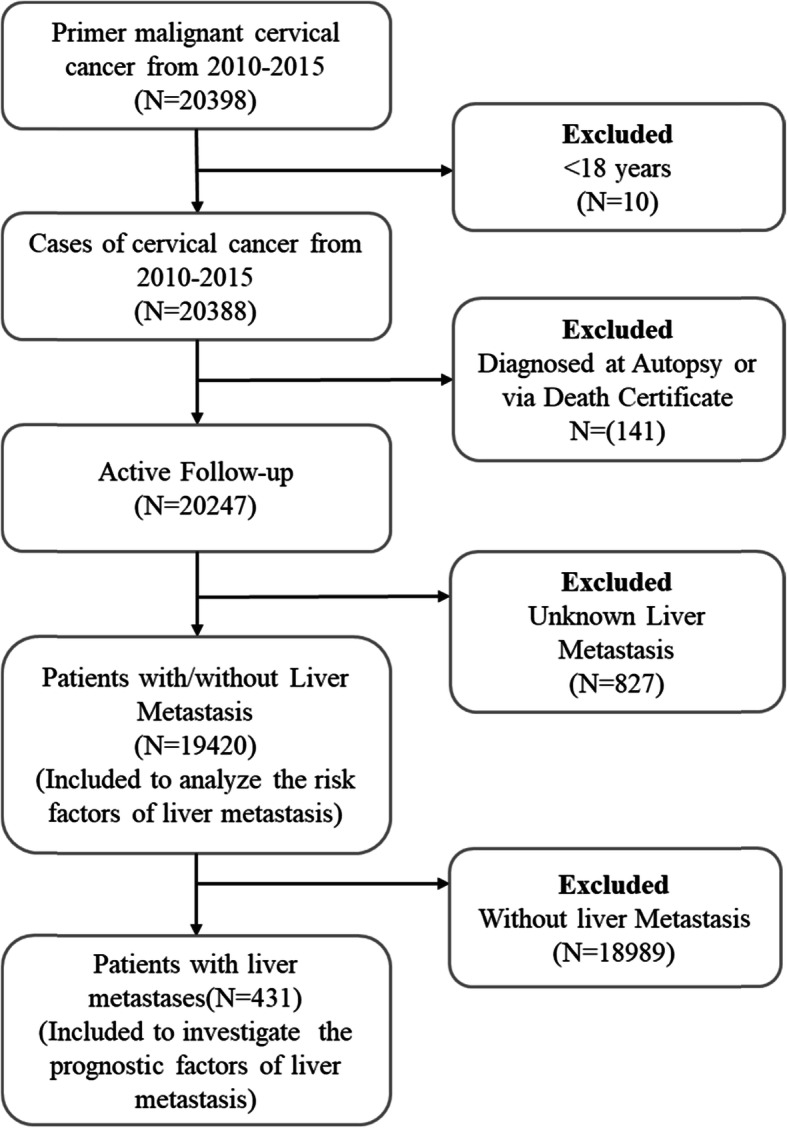
Flowchart of the cervical cancer patient’s selection in the present study

The data for this retrospective study was obtained from the Surveillance, Epidemiology and End Results (SEER) database 8.3.6 (http://www.seer.cancer.gov/seerstat). The surveyed SEER data consisted of 18 registries based on the 2010 census, covering approximately 27.8% of the cancer diagnosed population in the United States. The SEER office has authorized us to use the patient’s chemotherapy and radiotherapy records. According to the definition of site recording ICD-O-3/WHO 2008, the inclusion criteria in the primary site of “cervix and uterus” were selected. Since the metastatic status of the cancer was not recorded before 2010, the date was acquired for cases between January 1, 2010 and December 31, 2015. Exclusion criteria were as follows: type of diagnosis reporting source from autopsy or through death certificate, age at diagnosis less than 18 years old and unknown liver metastasis. The procedures for screening target patients were depicted in Fig. 1.

Demographic characteristics were collected: age at diagnosis, race, marital status, insurance status, primary site AJCC stage, grade, histological subtypes, tumor size, surgery of primary site, cancer metastasis, radiotherapy, chemotherapy and survival status. These were selected for further analysis for risk and/or prognosis factors in cervical cancer patients with liver metastasis. The major terminal points were overall survival (OS) and cause-specific survival (CSS). The CSS was obtained based on the parameter of the cause of death (COD) after excluding missing/unknown COD. Individuals who died of other reasons determined to be unrelated to cervical cancer were treated as alive.

### Statistical analysis

All statistical calculations were performed using SPSS (version 22.0, IBM Corporation, Chicago, IL, USA), and GraphPad Prism (version 6.0, GraphPad-Prism Software Inc., San Diego, CA, USA) was used to draw Kaplan-Meier survival plots. A Chi-square test was performed for categorical variables. The log-rank test was applied to OS and CSS data to analyze survival differences. Logistic regression was prepared to analyze risk factors, and Cox regression was conducted to distinguish prognostic factors, where univariate factors analysis with *P*-value < 0.1 were further introduced into a multivariate analysis model. A two-sided P-value < 0.05 was considered statistically significant.

## Results

### Demographic and clinical characteristics for liver metastasis

Based on the inclusion criteria, a total of 19,420 patients were diagnosed with cervical cancer between 2010 and 2015, including 431 (2.32%) with liver metastasis and 18,127 (97.68%) without liver metastasis. The average age of all cervical cancer patients with liver metastasis was 57.61 ± 14.30 years, while the overall cohort mean age was 50.53 ± 14.96 years. Detailed features of cervical cancer patients with and without liver metastasis cohorts were listed in Table 1. The following characteristics were different across patients with or without liver metastasis: age in diagnosed years (χ^2^ = 68.154, *P* < 0.001), race (χ^2^ = 17.867, *P* < 0.001), insurance status (χ^2^ = 6.827, *P* < 0.001), AJCC T (χ^2^ = 169.341, P < 0.001) and N (χ^2^ = 468.148, *P* < 0.001) stage, grade (χ^2^ = 615.394, *P* < 0.001), histology (χ^2^ = 132.849, *P* < 0.001), tumor size (χ^2^ = 152.343, *P* < 0.001), surgery of primary site (χ^2^ = 365.563, *P* < 0.001), other metastatic organs including lung (χ^2^ = 2397.509, *P* < 0.001), bone (χ^2^ = 1723.957, P < 0.001), brain (χ^2^ = 685.061, P < 0.001), radiotherapy (χ^2^ = 8.924, P < 0.001), chemotherapy (χ^2^ = 22.572, P < 0.001) and survival status (χ^2^ = 773.048, P < 0.001).

**Table 1:** Demographic and clinical characteristics for cervical cancer patients diagnosed with and without liver metastasis

Subject characteristics	No. of cervical cancer patients (2010–2015)		χ2	*P*-value
	With LM (*N* = 431, 2.32%)	Without LM (*N* = 18,127, 97.68%)		
**Age in years**			68.154	**< 0.001**
18–40	55 (12.8%)	5320 (28%)		
41–64	244 (56.6%)	10,125 (53.3%)		
≥ 65	132 (30.6%)	3544 (18.7%)		
**Year of diagnosis**			3.63	0.163
2010–2011	124 (28.8%)	6292 (33.1%)		
2012–2013	149 (32.6%)	6175 (32.5%)		
2014–2015	158 (36.7%)	6522 (34.35)		
**Race**			17.867	**0.001**
White	296 (68.7%)	14,170 (74.6%)		
Black	88 (20.4%)	2620 (13.8%)		
Others	46 (10.7%)	2005 (10.6%)		
Unknown	1 (0.2%)	194 (1.0%)		
**Marital status**			1.183	0.553
Married	263 (61.0%)	12,062 (63.5%)		
Unmarried	138 (32.0%)	5733 (30.2%)		
Unknown	30 (7.0%)	1194 (6.3%)		
**Insurance**			6.827	**0.033**
Insured	380 (88.2%)	17,242 (90.8%)		
Uninsured	41 (9.5%)	1220 (6.4%)		
Unknown	10 (2.3%)	527 (2.8%)		
**Primary Site**			20.689	**< 0.001**
Endocervix	48 (11.1%)	3590 (18.9%)		
Exocervix	3 (0.7%)	341 (1.8%)		
Overlapping lesion of cervix	7 (1.6%)	304 (1.6%)		
Cervix uteri, unspecified	373 (86.5%)	14,754 (77.7%)		
**AJCC stage**				
T stage			169.341	**< 0.001**
T1	59 (13.7%)	10,397 (54.8%)		
T2	73 (16.9%)	4170 (22.0%)		
T3	144 (33.4%)	2911 (15.3%)		
T4	61 (14.2%)	671 (3.5%)		
Unknown	94 (21.8%)	840 (4.4%)		
N stage			468.148	**< 0.001**
N0	124 (28.8%)	13,554 (71.4%)		
N1	218 (50.6%)	4622 (24.3%)		
Unknown	89 (20.6%)	813 (4.3%)		
**Grade**			615.394	**< 0.001**
I	4 (0.9%)	2141 (11.3%)		
II	54 (12.5%)	5940 (31.3%)		
III	175 (40.6%)	5353 (28.2%)		
IV	28 (6.5%)	417 (2.2%)		
Unknown	170 (39.4%)	5138 (27.1%)		
**Histology**			132.849	**< 0.001**
SCC	207 (48.0%)	12,429 (65.5%)		
AC	97 (22.5%)	4366 (23.05)		
Others	127 (29.5%)	2194 (11.6%)		
**Tumor size**			152.343	**< 0.001**
≤ 2 cm	12 (2.8%)	4168 (21.9%)		
> 2, ≤4 cm	31 (7.2%)	3031 (16.0%)		
> 4 cm	140 (39.4%)	5861 (30.9%)		
Unknown	218 (50.6%)	5929 (31.2%)		
**Surgery primary site**			365.563	**< 0.001**
Yes	386 (89.6%)	8243 (43.4%)		
No	45 (10.4%)	10,712 (56.4%)		
Unknown	0 (0.0%)	34 (0.2%)		
**Lung Met**			2397.509	**< 0.001**
No	204 (47.3%)	18,331 (96.5%)		
Yes	204 (47.3%)	622 (3.3%)		
Unknown	23 (5.3%)	36 (0.2%)		
**Bone Met**			1723.957	**< 0.001**
No	286 (66.4%)	18,634 (98.1%)		
Yes	128 (29.7%)	330 (1.7%)		
Unknown	17 (3.9%)	25 (0.1%)		
**Brain Met**			685.061	**< 0.001**
No	391 (90.7%)	18,911 (99.6%)		
Yes	18 (4.2%)	61 (0.3%)		
Unknown	22 (5.1%)	17 (0.1%)		
**Radiotherapy**			8.924	**0.003**
Yes	206 (47.8%)	10,451 (55.0%)		
No/ Unknown	225 (52.2%)	8538 (45.0%)		
**Chemotherapy**			22.572	**< 0.001**
Yes	263 (61.0%)	9390 (49.4%)		
No/Unknown	168 (39.0%)	9599 (50.6%)		
**Status (%)**			773.048	**< 0.001**
Alive	37 (8.6%)	13,467 (70.9%)		
Dead	394 (91.4%)	5522 (29.1)		
Median survival time(IQR)	5 (2–11)	29 (14–52)		

Notes: *LM*, liver metastasis; *Met*, Metastasis

### Associated risk factors for developing liver metastasis

We used univariate and multivariate logistic regressions to identify potential risk factors for acquired liver metastasis in cervical cancer patients (Table 2). In the univariable analysis, elderly diagnosed patients (more than 40 years old), black ethnicity, uninsured status, higher T and N stage, poorer pathological subgroup, adenocarcinoma and other histological classification, bigger tumor size, extrahepatic metastasis in lung, bone, and brain were potential risk factors, and primary site of surgery was identified as a protective factor. Multivariate logistic regression showed that black/white ethnicity (OR:1.469, 95% CI: 1.118–1.932, *P* < 0.001), T3/T1 stage (OR:1.703, 95% CI: 1.179–2.460, *P* = 0.005), T4/T1 stage (OR: 2.699, 95% CI: 1.741–4.185, *P* < 0.001), N1/N0 stage (OR: 1.650, 95% CI: 1.274–2.138, *P* < 0.001), Grade II/ Grade I (OR: 3.127, 95% CI: 1.094–8.935, *P* = 0.033), Grade III/ Grade I (OR: 5.509, 95% CI: 1.960–15.417, *P* < 0.001), Grade IV/ Grade I (OR: 7.639, 95% CI: 2.503–23.311, *P* < 0.001), adenocarcinoma/squamous histology (OR: 2.052, 95% CI: 1.544–2.727, *P* < 0.001), and other histology/ squamous histology (OR: 2.262, 95% CI: 1.716–2.982, *P* < 0.001), lung metastasis (OR: 7.272, 95% CI: 5.687–9.299, *P* < 0.001), bone metastasis (OR: 4.875, 95% CI: 3.679–6.460, *P* < 0.001), and brain metastasis (OR: 4.655, 95% CI: 1.770–12.245, *P* < 0.001) were associated with higher occurrence of liver metastasis. Interestingly, primary site of surgery (OR: 0.341, 95% CI: 0.235–0.493, P < 0.001) was still a protective feature for developing liver metastasis in cervical cancer patients. Age in years and tumor size did not show obvious differences (*P* > 0.05) in the multivariate logistic regression.

**Table 2:** Univariate and multivariate logistic regression analysis for the associated risk factors for developing liver metastasis in patients diagnosed with cervical cancer

Subject characteristics	Univariate analysis		Multivariate analysis	
	OR (95% CI)	*P*-value	OR (95% CI)	*P*-value
**Age in years**				
18–40	Ref	1.00	Ref	1.00
41–64	2.331 (1.736–3.129)	**< 0.001**	1.071 (0.772–1.486)	0.681
≥ 65	3.603 (2.623–4.949)	**< 0.001**	1.105 (0.770–1.586)	0.587
**Race**				
White	Ref	1.00	Ref	1.00
Black	1.608 (1.263–2.047)	**< 0.001**	1.469 (1.118–1.932)	**0.006**
Others	1.098 (0.802–1.504)	0.559	1.088 (0.757–1.565)	0.648
Unknown	NA	NA	NA	NA
**Insurance**				
Insured	Ref	1.00	Ref	1.00
Uninsured	1.525 (1.099–2.115)	**0.012**	1.632 (1.124–2.368)	**0.010**
Unknown	NA	NA	NA	NA
**T stage**				
T1	Ref	1.00	Ref	1.00
T2	3.085 (2.185–4.356)	**< 0.001**	1.216 (0.827–1.789)	0.320
T3	8.717 (6.421–11.835)	**< 0.001**	1.703 (1.179–2.460)	**0.005**
T4	16.020 (11.107–23.107)	**< 0.001**	2.699 (1.741–4.185)	**< 0.001**
Unknown	NA	NA	NA	NA
**N stage**				
N0	Ref	1.00	Ref	1.00
N1	5.156 (4.125–6.443)	**< 0.001**	1.650 (1.274–2.138)	**< 0.001**
Unknown	NA	NA	NA	NA
**Grade**				
I	Ref	1.00	Ref	1.00
II	4.866 (1.760–13.451)	**0.002**	3.127 (1.094–8.935)	**0.033**
III	17.498 (6.486–47.205)	**< 0.001**	5.509 (1.960–15.417)	**0.001**
IV	35.940 (12.541–103.001)	**< 0.001**	7.639 (2.503–23.311)	**< 0.001**
Unknown	NA	NA	NA	NA
**Histology**				
SCC	Ref	1.00	Ref	1.00
AC	1.334 (1.046–1.702)	**0.002**	2.052 (1.544–2.727)	**< 0.001**
Others	3.476 (2.774–4.355)	**< 0.001**	2.262 (1.716–2.982)	**< 0.001**
**Tumor size**				
≤ 2 cm	Ref	1.00	Ref	1.00
> 2, ≤4 cm	3.552 (1.821–6.928)	**< 0.001**	1.564 (0.764–3.202)	0.221
> 4 cm	10.075 (5.603–18.116)	**< 0.001**	1.601 (0.834–3.071)	0.157
Unknown	NA	NA	NA	NA
**Surgery primary site**				
No	Ref	1.00	Ref	1.00
yes	0.090 (0.066–0.122)	**< 0.001**	0.341 (0.235–0.493)	**< 0.001**
Unknown	NA	NA	NA	NA
**Lung Met**				
No	Ref	1.00	Ref	1.00
Yes	29.471 (23.892–36.353)	**< 0.001**	7.272 (5.687–9.299)	**< 0.001**
Unknown	NA	NA	NA	NA
**Bone Met**				
No	Ref	1.00	Ref	1.00
Yes	25.272 (19.976–31.971)	**< 0.001**	4.875 (3.679–6.460)	**< 0.001**
Unknown	NA	NA	NA	NA
**Brain Met**				
No	Ref	1.00	Ref	1.00
Yes	14.272 (8.357–24.373)	**< 0.001**	4.655 (1.770–12.245)	**0.002**
Unknown	NA	NA	NA	NA

Notes: *NA*, not available; *Met*, Metastasis

### Cox proportional hazards regression analysis

We conducted univariate and multivariate Cox regressions to distinguish underlying prognostic factors in OS (Table 3) and CSS (Table 4) amongst cervical cancer patients with or without liver metastasis. In univariate analysis of the OS cohort, we found that patients with age higher than 65 years old, lung and bone metastasis, those without radiotherapy or chemotherapy showed differences in survival rate. However, multivariable Cox regression analysis evidenced that lung metastasis (OR:1.451, 95% CI: 1.175–1.793, *P* = 0.001), those without radiotherapy (OR: 1.555, 95% CI: 1.262–1.915, *P* < 0.001), or chemotherapy (OR: 3.312, 95% CI: 2.654–4.134, *P* < 0.001) were predictors of poor prognosis. In the univariate analysis of the CSS cohort, non-surgery primary site, lung and bone metastasis, and those without chemotherapy showed differences in survival rate, similar to the OS population. Multivariable Cox regressions revealed that lung metastasis (OR: 1.471, 95% CI: 1.171–1.847, *P* = 0.001), and those without chemotherapy (OR: 3.229, 95% CI: 2.551–4.086, *P* < 0.001) had worse outcomes. Lung metastasis and patients without chemotherapy had poor prognosis in both OS and CSS cohorts.

**Table 3:** Univariable and multivariable Cox regression analysis of overall survival in cervical cancer patients with hepatic metastasis in SEER database (2010–2015)

Subject characteristics	Univariable		Multivariable	
	HR (95% CI)	*P*-value	HR (95% CI)	*P*-value
**Age in years**				
18–40	Ref	1.00	Ref	1.00
41–64	1.244 (0.910–1.699)	0.171	1.207 (0.872–1.672)	0.257
≥ 65	1.402 (1.002–1.961)	**0.048**	1.253 (0.886–1.771)	0.202
**Surgery primary site**				
No	Ref	1.00	Ref	1.00
yes	0.668 (0.481–0.927)	**0.016**	0.716 (0.506–1.012)	0.058
**Lung Met**				
No	Ref	1.00	Ref	1.00
Yes	1.384 (1.128–1.698)	**0.002**	1.451 (1.175–1.793)	**0.001**
**Bone Met**				
No	Ref	1.00	Ref	1.00
Yes	1.244 (1.000–1.549)	**0.050**	1.160 (0.929–1.448)	0.190
**Radiotherapy**				
Yes	Ref	1.00	Ref	1.00
No/ Unknown	1.583 (1.297–1.933)	**< 0.001**	1.555 (1.262–1.915)	**< 0.001**
**Chemotherapy**				
Yes	Ref	1.00	Ref	1.00
No/Unknown	3.390 (2.738–4.198)	**< 0.001**	3.312 (2.654–4.134)	**< 0.001**

Notes: *Met*, Metastasis

**Table 4:** Univariable and multivariable Cox regression analysis of cause-specific survival in cervical cancer patients with hepatic metastasis in SEER database (2010–2015)

Subject characteristics	Univariable		Multivariable	
	HR (95% CI)	*P*-value	HR (95% CI)	*P*-value
**Age in years**				
18–40	Ref	1.00	Ref	1.00
41–64	1.311 (0.945–1.817)	0.105	1.189 (0.845–1.673)	0.321
≥ 65	1.373 (0.959–1.966)	0.083	1.128 (0.780–1.633)	0.522
**Surgery primary site**				
No	Ref	1.00	Ref	1.00
yes	0.687 (0.474–0.994)	**0.046**	0.821 (0.557–1.210)	0.319
**Lung Met**				
No	Ref	1.00	Ref	1.00
Yes	1.416 (1.136–1.765)	**0.002**	1.471 (1.171–1.847)	**0.001**
**Bone Met**				
No	Ref	1.00	Ref	1.00
Yes	1.240 (0.982–1.564)	**0.070**	1.114 (0.880–1.410)	0.370
**Chemotherapy**				
Yes	Ref	1.00	Ref	1.00
No/Unknown	3.117 (2.475–3.924)	**< 0.001**	3.229 (2.551–4.086)	**< 0.001**

Notes: *Met*, Metastasis

**Fig. 2:**
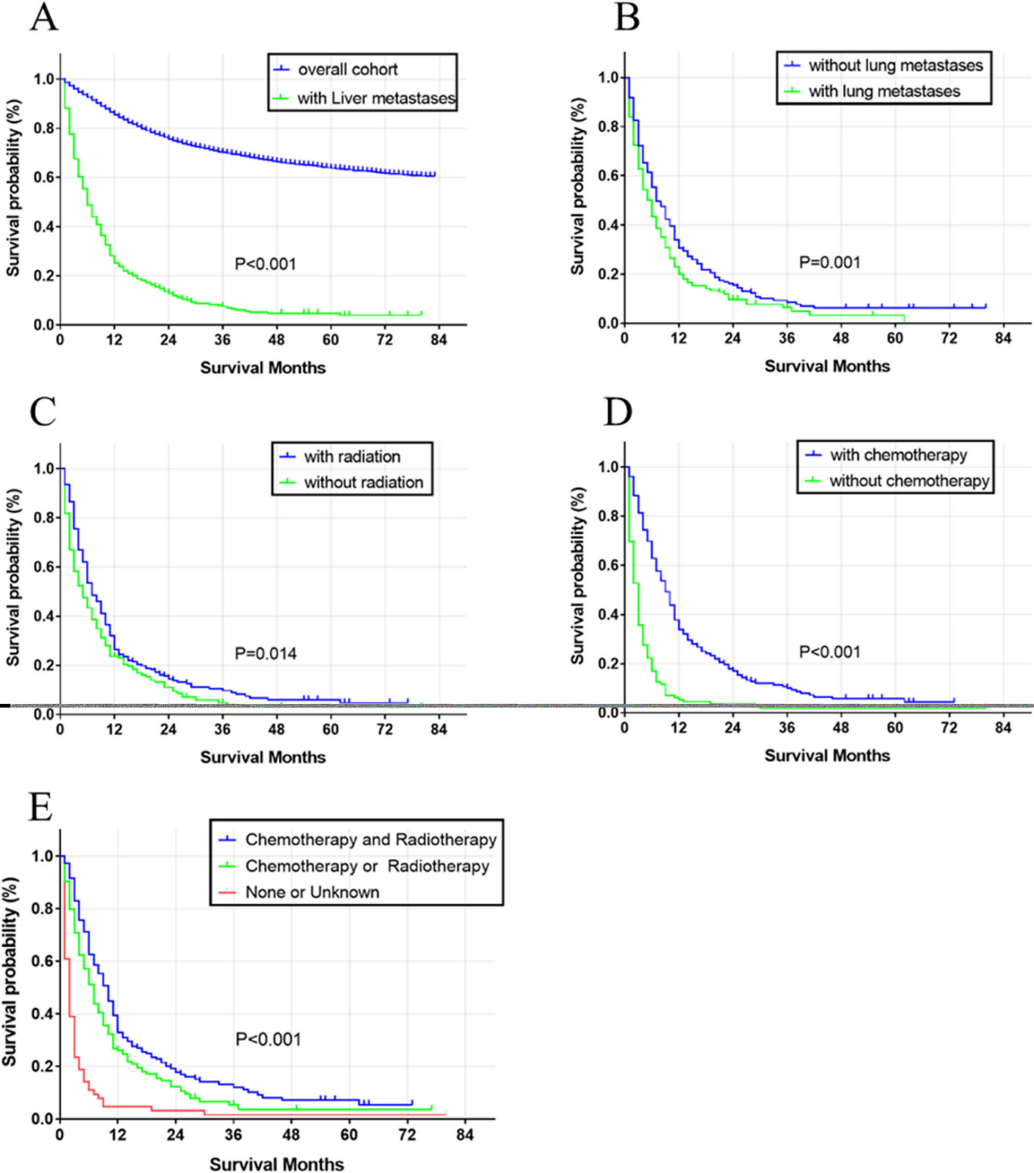
Overall survival among cervical cancer patients with liver metastasis. Overall cohort and with liver metastasis cohort (**a**). Stratified by lung metastasis (**b**), radiotherapy (**c**), chemotherapy (**d**), combined radiotherapy and chemotherapy or alone or not (**e**)

**Fig. 3:**
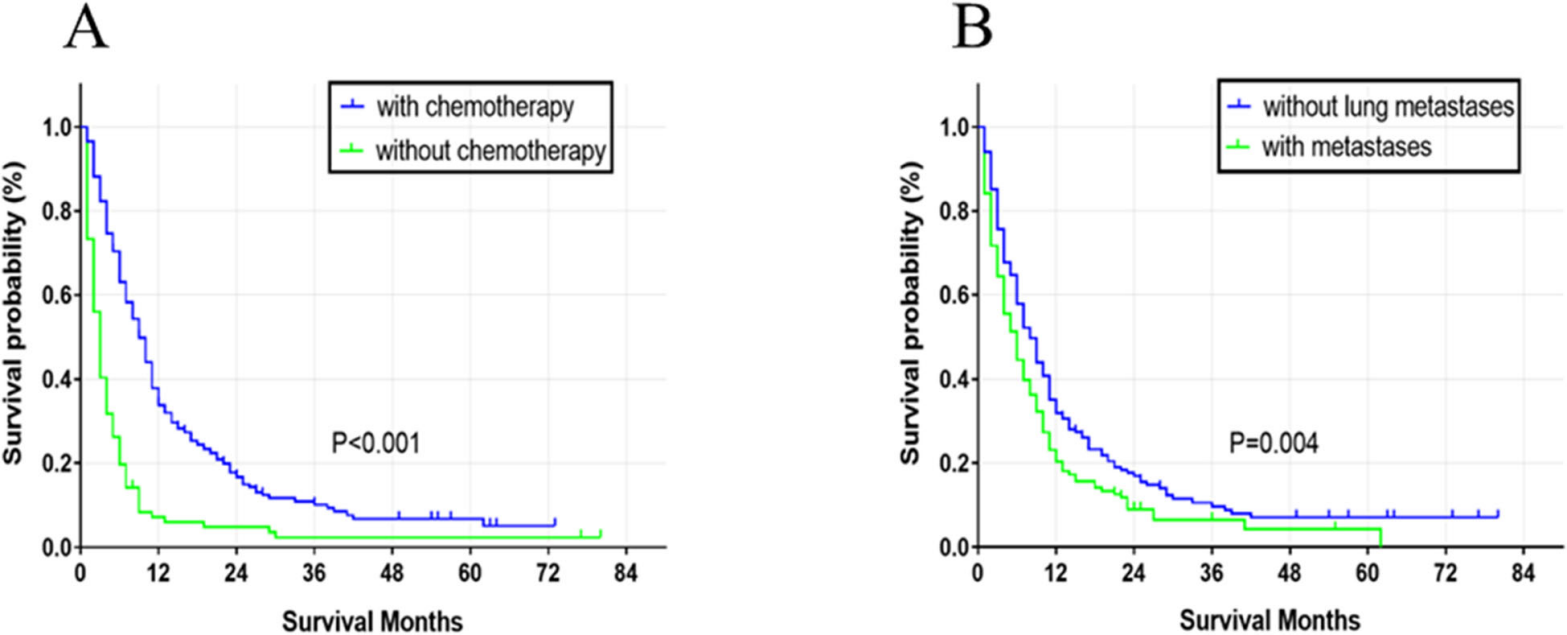
Cause-specific survival among cervical cancer patients with liver metastasis. Stratified by chemotherapy (**a**), lung metastasis (**b**)

OS survival curves of log-rank *P* < 0.5 factors are showed in Fig. 2. Compared to cervical cancer patients without liver metastasis, the median survival months (5 (IQR: 2–11) vs. 29 (IQR: 14–52), *P* < 0.001, Fig. 2a) greatly decreased in patients with liver metastasis. Among other potential prognostic factors for cervical cancer patients with hepatic metastasis, those without/with lung metastasis (6 (IQR: 2–13) vs. 4 (IQR: 1–9.75), *P* < 0.001, Fig. 2b) or with/without radiotherapy (7 (IQR: 3–13) vs. 3 (IQR: 1–8.5), *P* = 0.014, Fig. 2c) or with/without chemotherapy (9 (IQR: 4–16) vs. 1 (IQR: 0–3), *P* < 0.001, Fig. 2d) presented better survival curves. The combination of radiotherapy and chemotherapy led to an increase in survival when compared to those that received either radiotherapy or chemotherapy (10 (IQR: 4.5–17) vs. 6 (IQR: 3–11), *P* < 0.001, Fig. 2e), and patients that received no treatment (10 (IQR: 4.5–17) vs. 1 (IQR: 0–2), *P* < 0.001, Fig. 2e). CSS survival curves of log-rank *P* < 0.5 factors were drawn and presented in Fig. 3. Only patients with/without lung metastasis (4 (IQR: 1–10) vs. 6 (IQR: 2–13), *P* < 0.001, Fig. 3a) or without/with chemotherapy (7 (IQR: 3–13) vs. 3 (IQR: 1–9), *P* < 0.001, Fig. 3b) exhibited better survival time.

### Risk and prognostic factors for different metastases

**Fig. 4:**
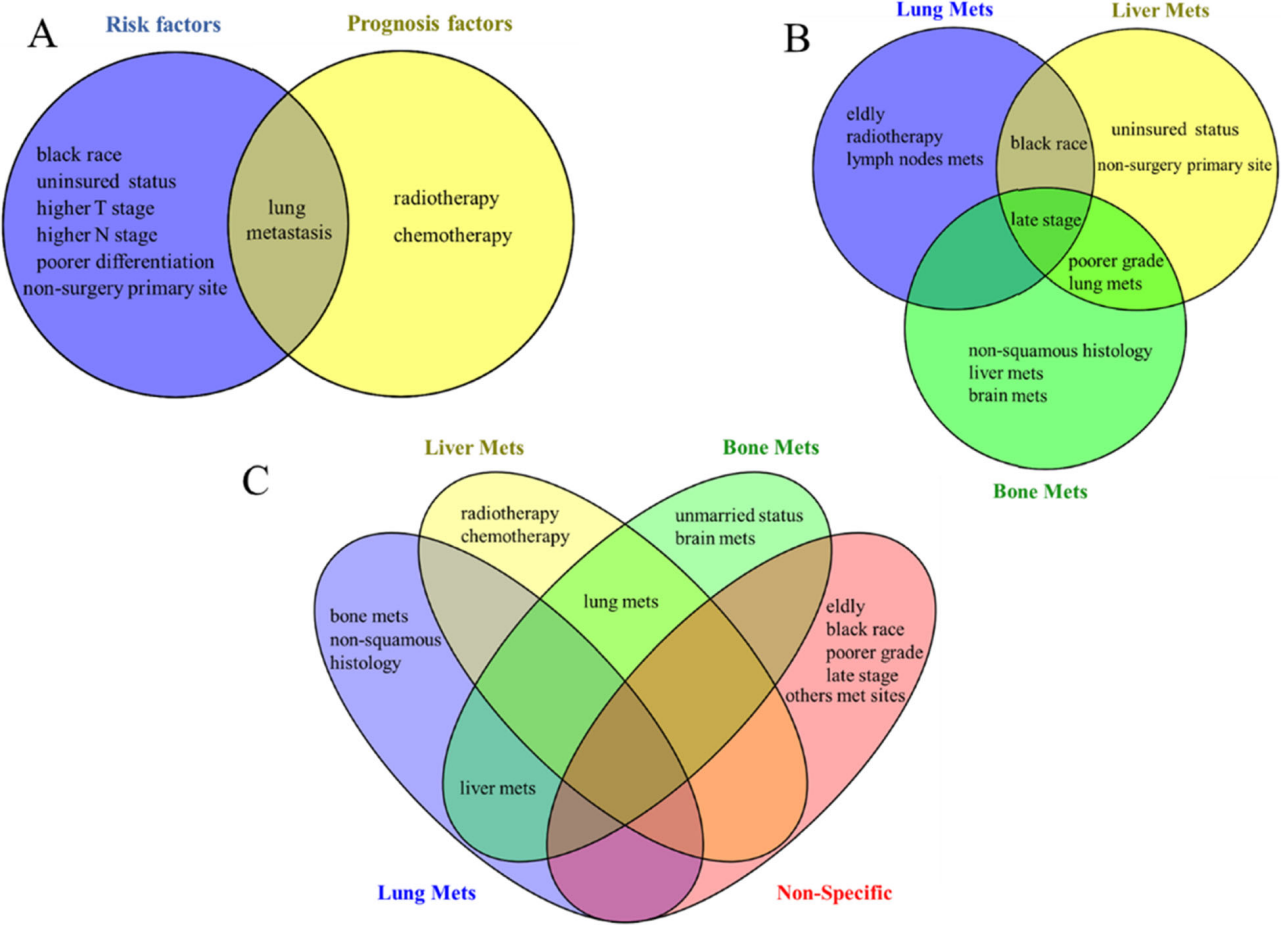
The Venn diagram of risk and prognostic factors of cervical cancer with hepatic metastasis in the current study, and common and independent risk and prognostic factors compared with other metastatic sites. **a** risk and prognostic factors of cervical cancer liver metastasis in this study, **b** risk factors of cervical cancer with lung, liver and bone metastasis. **c** prognostic factors of cervical cancer with lung, liver and bone metastasis

Independent and common risk and prognostic factors for cervical cancer patients with hepatic metastasis are listed in Fig. 4a. Lung metastasis was a unique common risk and prognostic factor. To uncover clinical-specific or common risk and prognostic factors for different metastatic sites among the cervical cancer population, we collected and compared clinical factors that were associated with cervical cancer with distant metastases, based on previous studies using the SEER database [5, 6, 8, 9, 11]. Late tumor stage was considered as a common potential risk factor for different metastatic sites. Poor grade and lung metastasis were regarded as shared risk factors for cervical cancer patients with liver and bone metastasis, while the black race was a unique risk factor for both liver and lung metastasis in cervical cancer patients (Fig. 4b). As shown in Fig. 4c, there was a lack of common prognosis factors for cervical cancer with different distant metastasis. Nevertheless, we found that lung metastasis was a common prognostic factor for cervical cancer with liver and bone metastases, while liver metastasis was a common prognostic factor for bone and lung metastases. For non-specific metastasis, any distant metastatic organ was identified as a potential prognostic factor, although the metastasis sites were not distinguished.

## Discussion

Advanced metastasis is one of the most important characteristics of cancers, accounting for a large proportion of cancer-associated deaths, in which complex biological profiles posed a huge challenge for clinical treatment. Since cervical cancer patients with liver metastasis present poor outcomes, there is a need to identify clinical risk and prognostic factors of hepatic metastasis in order to improve their survival condition. In this study, 2.32% (431/19420) of cervical cancer patients were diagnosed with liver metastasis, consistent with a previous study [5]. However, Kim GE et al. reported that 1.2% (20/1665) of cervical cancer patients were diagnosed with liver metastasis [12]. In another Chinese study, 0.99% (13/1312) stage IA2-IIB2 patients developed liver metastasis after radical hysterectomy [13]. The underlying reasons for this difference include the fact that our study was based on the population of the United States, and the inclusion of patients with hepatic metastasis diagnosed both during primary diagnosis or post-treatment.

In the present study, black ethnicity, uninsured status, higher tumor stage, poorer differentiated grade, and non-squamous histology were potential risk factors for liver metastasis in patients with cervical cancer. Black women showed higher incidence, metastasis, and mortality rate in many gynecological cancers, including ovarian, endometrial, and cervical cancers [14, 15]. This disparity of ethnicity might be due to the relatively lower socioeconomic status and health care system level for the black population, causing delayed diagnosis and treatment [16, 17]. The elevated risk of hepatic metastasis in cervical cancer patients with uninsured status supports this explanation. Additionally, adenocarcinoma and other non-squamous types of histological profiles were associated with hepatic metastasis in cervical cancer patients. A growing body of evidence suggests that cervical adenocarcinomas are more likely to induce distant invasion and less vulnerable to chemotherapy, possibly due to molecular alterations of somatic mutations, HPV integration, and gene expression [18–20]. Interestingly, primary site of surgery was identified as a protective parameter for liver metastasis in cervical cancer patients. This is probably because secondary liver metastasis occurs at a later time, thereby early surgical removal of the primary cervical lesions effectively preventing hepatic metastasis [21].

In our study, any lung, brain, and bone metastasis were significantly correlated to liver metastasis in cervical cancer patients. Several studies have described similar clinical phenomena: liver metastasis were shown to be accompanied by multiple metastatic organs [12, 13]. A possible explanation is that metachronous hepatic metastasis were more frequent than synchronous liver metastasis, resulting in longer intervals between the initiation of cervical cancer and the development of liver metastasis. On the other hand, early symptoms of liver metastasis are insidious, especially for isolated liver lesions, which are often asymptomatic. When liver symptoms appear, multiple extrahepatic metastases have usually occurred. Furthermore, the liver is irrigated by a rich blood supply from both arterial and portal venous systems, making hepatic metastatic cancer cells easier to spread to other distant sites through the bloodstream [22].

Patients with metastatic cervical cancer are considered incurable, but they can be treated to alleviate symptoms. Precancerous or stage IA cervical patients can be cured by surgical removal of the primary tumor, including trachelectomy or hysterectomy with/without lymph node dissection. Early-stage patients not suitable for operative treatment or stage IIB-IVA patients are more favorable for pelvic external beam radiotherapy (EBRT) and concurrent platinum-containing chemotherapy and brachytherapy. For stage IVB or distant metastases patients, platinum-based systemic chemotherapy combined with local-regional individualized radiotherapy is now the standard care in accordance with international guidelines [10, 23]. Our results corroborate this well-recognized treatment strategy. In this study, chemotherapy together with radiotherapy showed a better prognosis than radiotherapy or chemotherapy alone, and a superior advantage over no intervention in cervical cancer patients with liver metastasis. However, some patients with distant metastases rapidly evolved resistance or did not tolerate the toxic effects of chemotherapy. These patients were followed-up to receive chemoradiation combined with systemic bevacizumab treatment or pembrolizumab immunotherapy and presented better therapeutic effects [24, 25]. Additionally, the elimination of isolated liver metastasis was shown to prolong the survival time of a small number of patients [26, 27]. However, the majority of liver metastatic patients presented multiple extrahepatic metastases, therefore were not eligible for major hepatectomy. Altogether, cervical cancer patients with liver metastatic urgently require multi-model and individualized treatments to enhance therapeutic response and extend survival.

Cervical cancer patients that presented both liver and lung metastases had a poor prognosis. It was not surprising that patients with multiple organ metastases had worse outcomes than single-site metastasis, which has been confirmed in many metastatic malignant tumors [6, 11]. Considering that cervical cancer patients with different metastatic organs might benefit from individual treatment strategies [28], there are still scarce clinical risk and prognostic factors to predict the location and survival of different metastases. We found that lung metastasis was an independent indicator of risk and prognosis in hepatic metastasis in cervical cancer patients. This suggested that suspected liver metastasis in cervical cancer patients should simultaneously screen for other metastases by using effective imaging tools such as whole-body PET-CT or PET-MRI [29]. In collating the findings together based on the SEER database of different cervical cancer metastases, late tumor stage was the common potential risk factor for different metastatic sites, because the current staging standard of cervical cancer was based on the International Federation of Gynecology and Obstetrics (FIGO) Surgical Staging. Recently, a new Silva pattern system was introduced and considered to have a better prognostic value for endocervical adenocarcinoma [30, 31]. This could be further investigated for the clinical significance for distant metastasis. Our results showed lung metastasis was a prognostic factor for cervical cancer patients with hepatic and bone metastasis, while liver metastasis was a prognostic factor for patients with bone and lung metastasis. These associated clinical features can provide clues to the potential risks and prognosis of different distant metastasis in cervical cancer patients. More effective and precise clinical and molecular biomarkers need to be identified to help physicians to provide early diagnosis and treatment for metastatic cervical cancer patients [32].

There were several limitations to our study. First, many other clinical records were not included such as the sequence of distant metastatic organs and details of local metastases outside the pelvis. Second, we did not exclude patients followed-up for less than one year, because our preliminary results showed that the medium survival months of cervical patients with hepatic metastasis was less than one year. Finally, we did not have a validation cohort to confirm our findings, because the population of such patients was relatively small.

## Conclusion

Altogether, patients with cervical cancer rarely develop liver metastasis. Our results found some potential clinical characteristics that may be used to assess the risks and prognosis of liver metastasis in patients with cervical cancer. Chemotherapy combined with radiotherapy may be a suitable treatment strategy to improve survival time. These hepatic metastatic cervical cancer patients should be paid more attention to the risks and outcomes of other extrahepatic distant metastases. More specific and effective clinical and genetic biomarkers are needed to be established for the early detection and treatment of hepatic metastatic cervical patients, and ultimately enhance their survival time and quality of life.

## Data Availability

The data for this study extracted from Surveillance, Epidemiology and End Results (SEER) database 8.3.6 (http://www.seer.cancer.gov/seerstat).
